# Fostering innovation at Academic Medical Centers: The Case of University of Colorado Anschutz Medical Campus

**DOI:** 10.1017/cts.2021.792

**Published:** 2021-05-17

**Authors:** Cigdem H. Benam, Gali Baler, Richard Duke, Demetria M. McNeal, Kimberly A. Muller, Cathy Bodine, Elaine H. Morrato, Ronald J. Sokol

**Affiliations:** 1 CU Innovations, University of Colorado Anschutz Medical Campus, Aurora, CO, USA; 2 Division of Medical Oncology, Department of Medicine, University of Colorado Anschutz Medical Campus, Aurora, CO, USA; 3 Divison of General Internal Medicine, Department of Medicine, University of Colorado Anschutz Medical Campus, Aurora, CO, USA; 4 Colorado Clinical and Translational Sciences Institute, University of Colorado Anschutz Medical Campus, Aurora, CO, USA; 5 Department of Bioengineering, College of Engineering, Design and Computing, University of Colorado Denver, Aurora, CO, USA; 6 Center for Inclusive Design and Engineering (CIDE), College of Engineering, Design and Computing, University of Colorado Denver, Aurora, CO, USA; 7 Parkinson School of Health Sciences and Public Health, Loyola University Chicago, Chicago, IL, USA; 8 Department of Health Systems, Management and Policy, Colorado School of Public Health, University of Colorado Anschutz Medical Campus, Aurora, CO, USA; 9 Section of Pediatric Gastroenterology, Hepatology and Nutrition and the Digestive Health Institute, University of Colorado School of Medicine, Children’s Hospital Colorado, Aurora, CO, USA

**Keywords:** Innovation, commercialization, clinical and translational science, technology transfer, accelerator, University of Colorado

## Abstract

Commercializing biomedical discoveries is a challenging process for many reasons. However, Academic Medical Centers (AMC) that have teaching, patient care, research, and service engrained in their mission are well poised to host these discoveries. These academic discoveries can lead to improvement in patient health and economic development if supported to cross the “valley of death” through institutional assistance, by providing guidance, gap funding and product development expertise. Colorado has a vibrant local startup ecosystem, state support for commercialization and entrepreneurship as well as critical mass of product development expertise. University of Colorado Anschutz Medical Campus, as a major AMC, is an engine for growth for the region. This article discusses innovation efforts at the University of Colorado Anschutz Medical Campus as a case study, which is built around two major efforts: the CCTSI and CU Innovations. I-Corps at CCTSI and the SPARK|REACH program of CU Innovations have been instrumental in fostering innovation, commercialization, and entrepreneurship on the campus.

## Introduction

Academic medical centers (AMCs) have a well-established multipronged mission: patient care, education, research and engagement, and service to their communities which are intertwined in sophisticated ways. Faculty at AMCs produce scholarly products that report on biomedical and healthcare advances upon which scientific progress and education is built [1]. The funding that underpins these creative efforts is primarily reliant on federal sources and clinical income [1]. However, as a result of rising clinical costs, changing clinical payment methods, and the relative decline of governmental funding for research, the balance between the various missions is being tested and some believe the very survival of AMCs is at stake [2]. Innovation and commercialization of academic discoveries is being embraced as one way to meet these challenges, necessitating development of new ways to support faculty innovations and building new partnerships between administrative and academic units and industry. AMCs are also well poised, through innovation, to develop and disseminate creative solutions to the many challenges facing the U.S. healthcare system as well as competing economically in a global market.

The University of Colorado (CU) Anschutz Medical Campus is the largest AMC in the Rocky Mountain region. Since its opening in 2004, the two-hundred and thirty acre campus now includes more than eleven million square feet of state-of-the-art facilities where world class biomedical research, clinical care, and educational programs are delivered. Each year more than two-million patients receive care on the campus, at one of the three co-located health systems. The campus consistently receives over a half billion dollars of extramural funding for research, has 5,000 faculty, 300 post-doctoral fellows and 1,500 professional research associates actively working to contribute to scientific endeavors [3]. This cohabitation of clinical, research, and educational programs has afforded a vibrant collaborative culture centered around biomedical research and innovation. However, Anschutz Medical Campus is not immune to challenges AMCs are facing, nor is Colorado to the global pressures of economic competition. Hence, leadership of the campus has been strategizing on ways to foster academic innovations both to further the mission of the campus and to help alleviate chronic challenges of U.S. healthcare through “fill[ing] real gaps in science, medicine and healthcare with real products” [4]. This article describes broader efforts to expand commercialization of ideas generated on the campus into products and the infrastructure recently built to support translational work at this AMC. It will share challenges, best practices, and lessons learned with the hope of disseminating knowledge among other Clinical Translational Science Award (CTSA) programs and AMCs.

## The History of Innovation at the CU Anschutz Medical Campus

Commercialization of academic discoveries is challenging. There are numerous barriers that prevent rapid translation of discoveries into products. First is the skills gap. Academic investigators are trained in hypothesis-driven research and are not experienced in identifying commercial potential nor commercialization pathway. They lack the knowledge and understanding about how new technologies are brought to market. Second is lack of access to technology development expertise or commercialization resources required for early stage product development. Access to regulatory, reimbursement, and product development experts among others is vital for success but quite difficult to find at AMCs. Third, moving from basic research discoveries to scientific proof of feasibility or validation studies is typically not supported by traditional NIH grants and thus requires gap funding. In many instances, investigators do not have access to such funds [5]. The infamous “valley of death” (VoD) that is embodied by this funding gap is a major challenge for any academic product development exercise. Most of the time, academic discoveries are too early for industry partners who have the necessary capital, product development expertise, and infrastructure to bring a medical product to market. Saguy (2011) recommends, to bridge VoD, academia should actively work to reach out to industry as early as possible.

Conducting and excelling in basic and fundamental research is a prerequisite. Crossing the VoD by learning industry’s needs and driving inventions at least past the pre-NPD [New Product Development] stage, until the industry can pick them up, is also paramount. The typical pre-NPD includes four steps: 1. Affirming the technical viability of the invention as a product or service; 2. Formalizing the product concept; 3. Validating the concept with market research; 4. Developing a business case to gain commercial support, again using consumer research and marketing [6].

The University and Small Business Patent Procedures Act of 1980, or what is commonly known as the Bayh-Dole Act, “designed to accelerate the commercialization of technologies, gave universities the right to claim ownership to inventions within their institutions that were supported by federal funds” [1]. It led to the formation and proliferation of technology transfer offices at AMCs across the U.S. During the last four decades, the scope of work of technology transfer offices has evolved. Although initially focused on capturing intellectual property (IP), it soon became evident that IP is only a portion of this arduous process [7]. A major prerequisite for a successful technology transfer organization is understanding the needs of industry *and* the market. In turn, this requires interdisciplinary teams working together both assessing the viability of the invention and developing it as a product. Technology transfer officials generally have IP knowledge with a scientific background and do not have the human capital to deliver in all these domains. Hence, universities have been building innovation offices and programs to bring critical commercialization and business development expertise in house [8]. CU Anschutz is one of them. Over the past decade, the campus has been undergoing a transformation through identifying resources to facilitate the four steps of pre-NPD that Saguy highlights.

Building on its strengths in performing cutting edge basic research, deep understanding of biology, disease modeling, and clinical mechanistic studies over the years, CU Anschutz has invested in talent and infrastructure for translational research, while cultivating a proentrepreneurial culture. Catalyzing this transformation has been the awarding of numerous grants and philanthropic donations to support translational research, including the creation of the Colorado Clinical and Translational Sciences Institute (CCTSI) in 2008 funded by the CTSA Program of the National Center for Advancing Translational Sciences (NCATS), CU Comprehensive Cancer Center grants from the National Cancer Institute, and the CU Gates Center for Regenerative Medicine, to name a few. A number of groundbreaking CU Anschutz discoveries led to major improvements in patient care and public health. Two vaccines for shingles, Zostavax and Shingrix, are examples of blockbuster drug discoveries that were discovered at CU Anschutz, similar to Kineret for rheumatoid arthritis and Botox for hyperactive bladder. Myogen, Synergen, Taligen, GlobeImmune, and miRagen are examples of start-ups that underwent major acquisitions, the first two being acquired by two major pharma companies for $2.5 billion and $1.1 billion, respectively. Despite these successes, certain challenges prevail. Acknowledging and eliminating roadblocks to innovation and commercialization, the university restructured its technology transfer office, now called CU Innovations, in 2016 to adopt a fresh comprehensive perspective enriched with best practices.

CU Innovations has been working in collaboration with academic and administrative offices of the university to identify unique barriers for translation and find creative ways to partner with industry and fill in skills gap for commercialization. At its core lies the traditional patent licensing and patent management. However, in recent years, greater emphasis has been placed on industry collaborations, providing gap funding, making technology development experts accessible to faculty, and offering training programs. Technology development, startup formation, business development, and venture development are also within the remit of CU Innovations. The office hired accomplished Entrepreneurs in Residence to help faculty in maturing their commercialization ideas and setting up ventures. Finally, what might not exist in many other innovation offices is the close collaboration with hospital partners on the campus for healthcare innovations through codevelopment of technologies emerging both from within the campus and outside. This partnership utilizes the offerings of the 4^th^ Industrial Revolution to make a change in the US healthcare system by collaborating with startups to multinational industry partners.

**Fig. 1.**
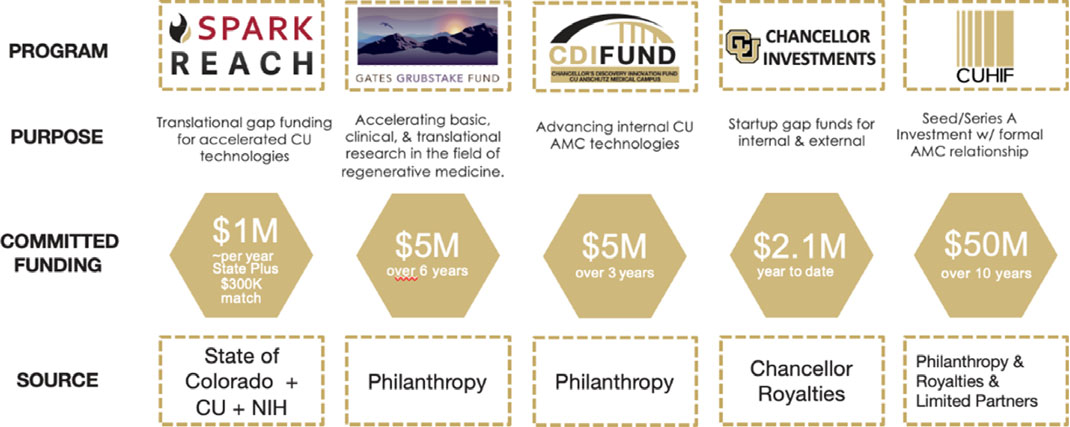
CU Anschutz Medical Campus Commercialization Funding Mechanisms, Dollar Amounts and their Sources. CU: University of Colorado; REACH: Research Evaluation and Commercialization Hub; CDI Fund: Chancellor’s Discovery and Innovation Fund; CUHIF: University of Colorado Healthcare Innovation Fund.

Acknowledging the importance of gap funding, CU Innovations set up various funding mechanisms (Fig. 1). Two accelerator programs, SPARK Colorado and Gates Grubstake, were established to focus solely on proof-of-concept work. Another mechanism created is the Chancellor’s Discovery and Innovation Fund providing milestone-driven funding for certain key experiments to help unlock further funding. It also acts as a pipeline for the two accelerator programs on the campus. Finally, the university’s $50 million investment fund provides flexibility to invest in both internal and outside technologies.

The CCTSI has been a major partner of CU Innovations as it is designed to improve and streamline the translational research process and to catalyze innovation in the training of translational scientists and the development of new research tools [1]. Its work spans across translational workforce development to building a robust innovation ecosystem and demonstrating clinical and translational impact.

The trained clinician or translational research scientist often has little knowledge about how to advance solutions from concept to commercial value. To support this workforce development need, Colorado was one of the 10 CTSAs selected nationally by NCATS to adopt the Innovation Corps™ (I-Corps™) program for clinical and translational researchers [9]. I-Corps is an accelerated version of the Stanford University’s Lean Launchpad course [10,11] originally developed in partnership with the National Science Foundation and expanded to other federal agencies. The U.S. Department of Commerce has reported on the success of I-Corps in preparing scientists, engineers, and graduate students to extend their focus beyond the academic campus for greater societal impact [12].

Locally identified as I-Corps@CCTSI, the program has established a clear path for CU AMC scholars to translate research innovation and discoveries into commercially viable products or services. This team-based experiential three-week training is taught by faculty with entrepreneurial experience. The objectives of the I-Corps program are to a) develop the workforce by catalyzing an academic entrepreneurial culture and skillset; b) develop discoveries and commercialization potential, and, c) demonstrate impact by connecting researchers to resources for commercialization, domain expertise, and accelerator funding.

A defining feature of the I-Corps program is the customer discovery process [13,14]; a customer-centric approach to determine if there are actual customers for a product/service to ensure product-market fit. During the customer discovery process, participants take on the role of an empirical detective, allowing evidence to lead them to a solution without letting any bias get in the way. Through this process, I-Corps equips teams to anticipate the barriers (e.g., adoption, market needs) surrounding early stage technologies and avoid VoD between research and development and commercialization.

**Fig. 2.**
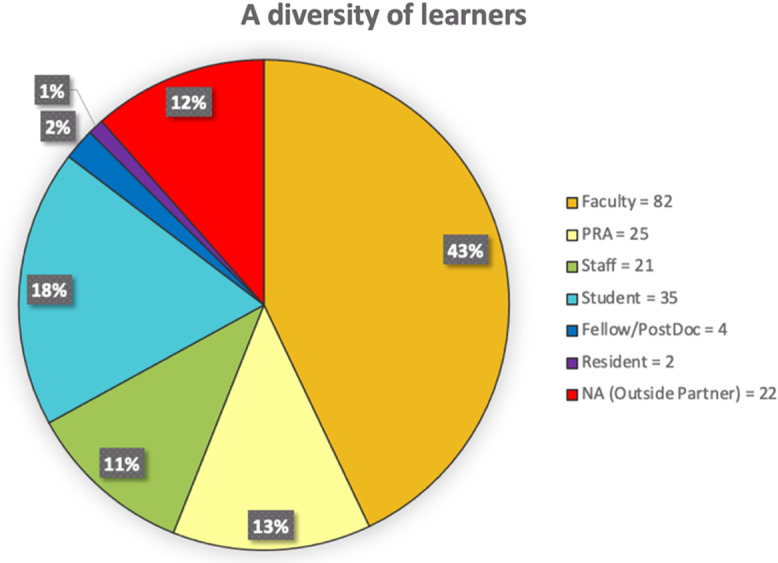
I-Corps@CCTSI Participants since 2016. I-Corps: Innovation Corps; CCTSI: Colorado Clinical and Translational Sciences Institute; PRA: Professonal Research Assistant.

Participating faculty and students use a business model canvas framework to conduct customer discovery, gather market force data, and gain a clear understanding of the value of their invention to the marketplace. From 2016 to fall of 2019, I-Corps@CCTSI has trained 8 cohorts, with 68 teams and 191 participants from diverse backgrounds (Fig. 2). The customer discovery interview process has resulted in 1,690 interviews to date (median=25 per team). Perhaps most importantly, teams have consistently reported immediate learning outcomes by describing their experience as “sharpened our pitch” and “helped me hone our product-market fit” from the the most recent Fall, 2020 cohort.

Most recently, I-Corps began offering financial seed grants to extend the customer discovery process, continue entrepreneurial momentum, and encourage commercialization.

Part of I-Corps’ mission is to synergize with other offerings of CCTSI, such as the pilot grant programs, other training programs, and clinical research units that support translational discoveries and to connect researchers to resources for commercialization mentorship, domain expertise, and accelerator funding. Therefore, I-Corps’ efforts and pilot programs are very complementary to CU Innovations’ efforts and indeed create a steady pipeline to the accelerator programs on campus. Most recently, this partnership between CU Innovations and CCTSI has culminated in receiving a National Institutes of Health (NIH) Research Evaluation and Commercialization Hub (REACH) Award in 2019. REACH at CU-Anschutz is built on the previous SPARK Colorado program that has been engaging with local ecosystem partners in creative ways and helping faculty translate their discoveries.

## Accelerating Treatments through SPARK | REACH Program

In 2018, the campus initiated a pilot accelerator program in collaboration with Stanford University, called SPARK Colorado. CU Anschutz preferred to adopt the proven model of Stanford for supporting faculty innovations and maturing inventions through the next inflection point where industry partners or investor groups would be willing to engage. SPARK Stanford was founded in 2006 around the time when the concept of accelerators was emerging in industry and has recorded a success rate of 51% of projects either licensed or entered the clinic. The program is built on three pillars: moderate funding, industry mentorship and, curriculum-based training on product development and commercialization.

The chief novelty of SPARK has been helping to bridge the cultural divide between academia and industry by creating venues for interaction and mutual learning. It has been derisking drug development, medical device, and diagnostic projects for outside companies, investors, and even for federal grant agencies. Many SPARK projects were not only licensed to outside companies with access to larger, needed funding but also were able to receive funding from NIH or Defense Advanced Research Projects (DARPA), after generating initial data supporting their hypothesis [15]. Another rewarding aspect of SPARK, from the perspective of universities, at a time of a shrinking academic job market, has been providing graduate students and post-doctoral fellows the skills necessary for industry jobs.

SPARK Colorado announced its call for applications on February 1, 2018, for its inaugural year. There were three major project selection criteria: scope of unmet medical need, novelty of the approach, and feasibility in terms of time and financial cost to move a project forward. SPARK Colorado, unlike Stanford’s single focus on drug development, accepts projects in drug development, medical devices, and diagnostics. It is a cohort-based program in which about 10 admitted teams per year meet on a bi-weekly cadence with voluntary mentors and other teams. They discuss progess in their programs or host a guest speaker on key topics such as regulatory affairs, reimbursement, intellectual property (IP), medicinal chemistry, or venture development. Most of these training sessions are open to everyone on campus. During its first two years of the program, over 1,000 individuals participated in the 45 SPARK Colorado seminars.

A major challenge for the entrepreneurial space is the availability of small business concerns with the appropriate expertise, organizational structure, and competence in commercialization. Colorado has a unique ecosystem for startups, which is to the advantage of the University of Colorado. Boulder, Colorado is a nationally renowned startup hub that has built a unique ecosystem particularly for technology companies. A major global accelerator program, Techstars, based in Boulder, was successful in building a tight-knit network of entrepreneurs and consultants for small businesses. They have been also successful in recruiting talent from across the nation. Bred Feld in his book *Startup Communities: How to Build an Entrepreneurial Ecosystem in Your City* describes in detail how the Boulder ecosystem was established in the 1990s and potentially how it can be replicated elsewhere and what would be the minimum requirements. In that sense, Colorado is relatively better off in tackling the challenges of entrepreneurial space as it has established components of an innovation ecosystem supporting startups. Yet, Colorado shares a restrain that many other inland states face, which is access to capital. Particularly many of the biotech companies move to east or west coast after or for raising funds from venture capital.

**Fig. 3.**
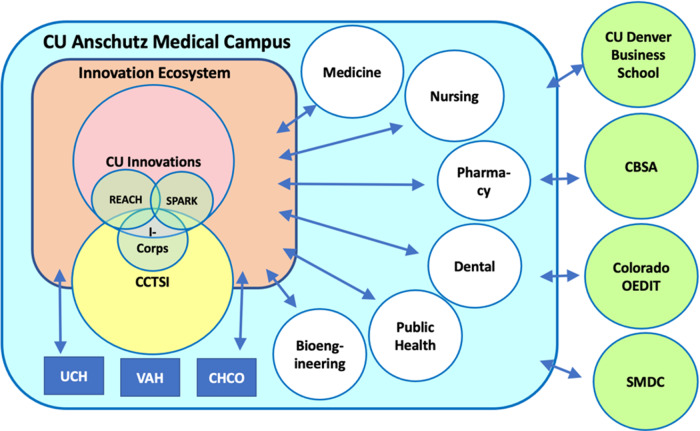
University of Colorado Anschutz Medical Campus Innovation Ecosystem. The ecosystem is composed of two core components, CU Innovations and CCTSI. Through initiatives that are run by each of these core components, i.e., I-Corps through CCTSI and SPARK| REACH through CU Innovations, they create synergies and interact with all the schools (i.e. Medicine, Nursing, Pharmacy, Dentistry, Public Health, and Department of Bioengineering) and affiliated hospitals on the campus as well as external partners. CU Innovations: Innovation office of the CU Anschutz Medical Campus; CCTSI: Colorado Clinical and Translational Sciences Institute; REACH: Research Evaluation and Commercialization Hub; UCH: University of Colorado Hospital; VAH:Veterans Affairs Hospital; CHCO: Children’s Hospital Colorado; CBSA: Colorado Bioscience Association; Colorado OEDIT: The State of Colorado Office of Economic Development and International Trade; SMDC: Small Business Development Centers.

Initiation of the SPARK program at CU Anschutz has enabled the campus to better engage with the local innovation ecosystem (Fig. 3). The program has been recruiting volunteer mentors from the established networks of CU Innovations, the Colorado Bioscience Association (CBSA), and the Fitzsimmons Innovation Community (FIC). CBSA is the major driver of the bioscience industry in Colorado with over 400 life science companies representing 16,000 employees. FIC manages over 300,000 square feet of biotechnology incubator space directly adjacent to the CU Anchutz campus and houses over 75 companies and startups as well as the Gates Biomanufacturing Facility.

Mentors come from diverse backgrounds including former executives in large drug development and medical device companies or entreporenurs who successfully launched and exited startups. The program has 30 mentors that engage with the teams at different levels and cadence. They assist faculty in developing and providing the information essential for externalizing their technology, such as defining a clear clinical indication, understanding the market and competitive landscape, navigating FDA regulations, developing a roadmap for first-in-human studies, and understanding the reimbursement landscape. All of these taken together build the economic value proposition for the product under development.

A critical component of SPARK Colorado is active project management to help teams establish project plans and ensure they follow timelines and deliverables. A project manager with the requisite experience in drug, device, or diagnostic development is assigned to each project to oversee the research effort and to assure that the project is tracking toward milestones that will increase the likelihood of creating a viable start-up company or partnering opportunity. SPARK project managers meet with teams on a regular basis and plug in resources such as regulatory consultants or Contract Research Organizations when necessary; they work with teams to identify commercialization strategy gaps and analyze the competitive landscape.

SPARK Colorado has a call for applications annually. Faculty apply through a digital platform to which external reviewers have access. This external review board is composed of the mentors of the program. Once written reviews are completed, in a second review phase, selected faculty are invited to a live pitch session. Project managers of the program work closely with faculty to hone their applications and their pitches. A major challenge for this kind of program at an academic institution is to identify the best projects as well as the teams that are most likely to execute on their ideas. SPARK Colorado has continued to work on improving its process for team and project selection with room still to grow.

The program has been experimenting with various models to cover costs. For the first year, funding for selected projects came from the faculty members’ departments, whereas programmatical expenses were covered by CU Innovations. In the second year, the program merged with the Advanced Industries Accelerator (AIA) funding of the State of Colorado, Office of Economic Development and International Trade (OEDIT). The State of Colorado has been funding innovation through technology transfer offices of research institutions including CU Anschutz by awarding grants for proof of concept projects. Universities are expected to provide matching funds in order to receive state dollars.

A major challenge for Colorado startups, similar to many other companies founded in inland states, is access to capital. Colorado does not have any restrictions in its law, unlike some other states, that hinder state dollars being spent to support private industries. Indeed, the State of Colorado has been instrumental in filling a major gap as it provides direct support to private industries through grants and tax credits. A major venue for this is the AIA program run by OEDIT, funded by tax revenues. This is a grant mechanism to support proof of concept work conducted within universities as well as early-stage companies in advanced manufacturing, aerospace, biosciences, electronics, energy and natural resources, infrastructure engineering, technology, and information. The state provides early-stage capital, collaborative infrastructure, and export grants as well as investment tax credit. All these are governed by the Procurement Code of the State of Colorado, which does not hinder the state in providing funding for private industries. The AIA program is funded through 2024 and has been critical in advancing life science innovations. This required collaboration between the Colorado Bioscience Association, the Office of the Governor of Colorado, and the research institutions.

## SPARK Transitioning to REACH

**Fig. 4.**
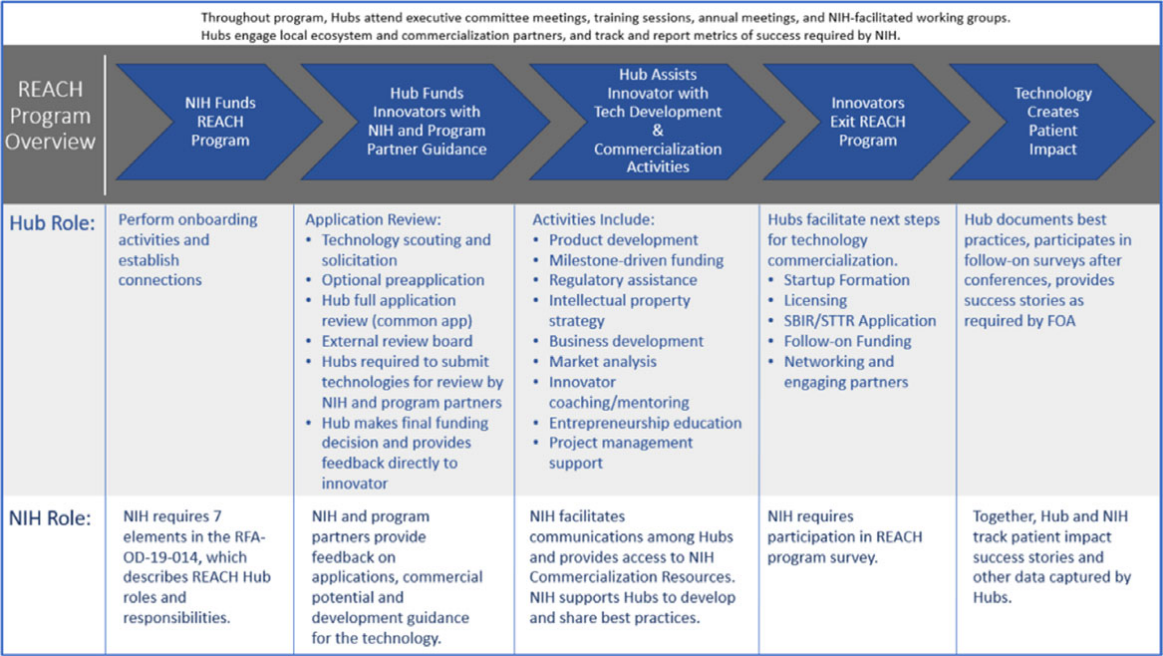
REACH Program Overview. Source: The Handbook: NIH Research Evaluation and Commercialization Hub (REACH) Stand-Up, November 26, 2019.

NIH has been reflecting on ways to translate basic research discoveries conducted with federal dollars to products to have broader impact of taxpayers’ money. With this goal in mind, NIH established the REACH program in 2013 (Fig. 4). Initially, it supported establishment of NIH Centers for Accelerated Innovations as a result of public–private partnerships in Boston, California, and Cleveland. In its second round of awards, NIH funded three REACH hubs in Minnesota, Long Island, and Louisville. Most recently in 2019, NHLBI/NIH funded five new REACH Hubs. Building on the success of SPARK Colorado, Anschutz Medical Campus has become one of these five newest hubs [16].

The Colorado REACH hub has three major goals: identifying the most promising technologies and providing funding for product definition studies; promoting commercialization of selected products by enabling access to technology development resources and facilitating formation of strong spin out companies; and expanding educational, experiential, and networking opportunities (Fig. 4). Colorado REACH has an innovative governance structure where seasoned faculty entrepreneurs, CCTSI and CU Innovations, are all represented on the Executive Committee responsible for the broader direction of the hub. The REACH program centralized funding sources and enabled efficient use of research and product development dollars.

A crucial component of biomedical innovation is navigating FDA regulations. As already mentioned, SPARK investigator teams are paired with mentors who generally have high-level knowledge of the regulatory process as many of them are accomplished entrepreneurs that have taken products to market. However, the program also requires the teams to work with a paid consultant to provide specific input pertaining to their projects. The funding that comes from the State of Colorado enabled teams to budget consultants for their projects. But most recently, the NIH REACH grant also allowed the program to set aside dedicated funds for consultants who are regulatory and reimbursement experts that work with teams as they start to define milestones to achieve during the program and afterwards. Finally, the university also has an office that facilitates investigator-initiated INDs on behalf of the university. However, since it has limited human resources, the office is generally quite selective in deciding which project to support.

Apart from grant dollars, REACH has provided the SPARK program with new resources provided by the proof of concept network. All of the projects that are selected for advancement after the initial review at CU Anschutz are also reviewed by a Technology Guidance Committee (TGC) consisting of experts assembled by the NIH, including an entpreneur-in-residence, a regulatory expert and representatives from the FDA, USPTO, CMS, Kaiser Permanente, and a liaison from the relevant institute at NIH. The TGC provides invaluable early input from key experts. The extra grant dollars also enabled the campus to put aside dedicated funds for paid consultants not only to serve the selected projects but also to the whole campus through office hours. REACH also provided a broader umbrella to build new partnerships between campuses of CU and beyond. Projects are encouraged to make use of expertise and resources available from our collaborating partners including the UC Cancer Center Developmental Therapeutics Program, Department of Bioengineering, CU Denver School of Business, School of Pharmaceutical Sciences, Colorado Office of Economic Development and International Trade (OEDIT), Rockies Venture Club, Small Business Development Centers (SMDC), and the Colorado Biosciences Association (CBSA).

A major pillar of REACH, similar to SPARK, is education and culture change. During the application process for the first REACH cohort, CU Anschutz worked with the CU Denver Business School in organizing a “Bio Bootcamp” to introduce fundamental concepts of business and product development. Similarly, MBA students engage with project teams in helping with market research or preparing commercialization plans. The CU Anschutz REACH team is working to build a robust educational component that is developing targeted and easily accessible material while also incorporating existing resources on the campus including the Bio-Design course in the Department of Biomedical Engineering, the Drug Development course in the School of Pharmacy, I-Corps program provided by the CCTSI, and the Colorado SPARK curriculum around commercialization to provide broader visibility and access.

In certain cases there can be challenges working with health systems in terms of distribution of faculty time between clinical activities and entrepreneurial activities. In the Anschutz case, UCHealth, Children’s Hospital Colorado and the University of Colorado are separate legal entities. Clinicians working at UCHealth and the Children’s Hospital are the faculty of the university and they work for the medical school. Therefore, the decisions about allocation of faculty effort and time dedicated to innovation and entrepreneurship is up to the departments and division chairs, and not to the hospital systems. Having said that, if faculty have extensive hours in clinic it might be challenging to dedicate extra time for their innovation and entrepreneurship related efforts. In these cases, faculty can buy out their clinical time through grants. The campus is currenlty engaged in a process to standardize institutional support for innovation and entrepreneurship for faculty through a committee working on revising tenure and promotion criteria. In its current form, there exists divergence between departments and divisions’ support for such activities.

## Conclusion

The University of Colorado, Anschutz Medical Campus as the largest academic healthcare center in the Rocky Mountain region has been investing in fostering innovation on multiple fronts. Having an innovation friendly and supportive leadership is essential to enable organizational culture change that will embrace innovation at various layers of the organization. Leadership must acknowledge that returns from such programs may take years to fully become realized. Restructuring the technology transfer office in 2016 and tasking it to identify institutional roadblocks for product development and interaction with industry was a major step forward. The close collaboration between CU Innovations and the CCTSI with a shared mission of supporting translation of discoveries had led to major successes, one of which is the NIH REACH award. SPARK Colorado and later REACH has enabled a growing community of innovators. The collective efforts of these disparate groups and the continued commitment of campus toward cultivating a culture of innovation have empowered faculty and students to make a direct impact to improve human health and created new venues for the campus to engage with the local ecosystem partners and broader community.

During the three years of SPARK| REACH program, in total it has received 155 applications, of which 28 projects were admitted to the program. In total, SPARK teams generated $11.1 million follow on funding as well as $2.9 million in investments. As a result of the program, 6 new patents were filed, 6 new startups were formed. But most importantly, SPARK| REACH in collaboration with the CCTSI has accelerated the change of culture on the campus, raising awareness and interest among faculty and students for commercialization and entrepreneurship.
